# GIS-based spatial modelling of COVID-19 death incidence in São Paulo, Brazil

**DOI:** 10.1177/0956247820963962

**Published:** 2021-04

**Authors:** Rodrigo Custodio Urban, Liane Yuri Kondo Nakada

**Keywords:** geographically weighted regression model, informal urban settlements, people per household, population density, SARS-CoV-2, São Paulo, spatial error model

## Abstract

Seeking to understand the socio-spatial behaviour of the COVID-19 virus in the most impacted area in Brazil, five spatial regression models were analysed to assess the disease distribution in the affected territory. Results obtained using the Spearman correlation test provided evidence for the correlation between COVID-19 death incidence and social aspects such as population density, average people per household, and informal urban settlements. More importantly, all analysed models using four selected explanatory variables have proven to represent at least 85 per cent of reported deaths at the district level. Overall, our results have demonstrated that the geographically weighted regression (GWR) model best explains the spatial distribution of COVID-19 in the city of São Paulo, highlighting the spatial aspects of the data. Spatial analysis has shown the spread of COVID-19 in areas with highly vulnerable populations. Our findings corroborate reports from the recent literature, pointing out the need for special attention in peripheral areas and informal settlements.

**Figure:**
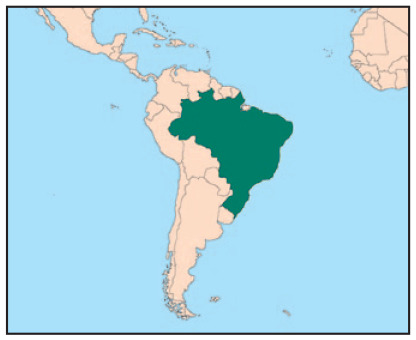


## I. Introduction

The World Health Organization (WHO) has recognized the new coronavirus as a pandemic,^(1)^ and as of 22 June 2020, SARS-CoV-2 has infected more than 9.1 million people worldwide, causing more than 473,000 deaths.^(2)^ Brazil was the second leading country in number of COVID-19 total cases and total deaths in June. São Paulo State accounted for more than 20 per cent of total confirmed cases in Brazil and the city of São Paulo for almost 50 per cent of total confirmed cases in São Paulo State.^(3)^

Understanding the incidence of COVID-19 is critical for effectively controlling the transmission. Therefore, the aim of this study was to conduct a GIS-based spatial modelling analysis of COVID-19 in the city of São Paulo, considering both environmental and social factors.

Spatial regression models have been recently reported as a useful approach for the assessment of the spatial distribution of SARS-CoV-2.^(4)^ While local models represent the spatial relationship among the variables, global models offer the advantage of a simple application for scenario assessment.

In the global models, the relationships between explanatory variables and dependent variables are assumed not to produce spatial variation.^(5)^ Ordinary least squares (OLS) is a regression model that evaluates the relationship between explanatory and dependent variables and does not consider the spatial dependency among observations. The spatial lag model (SLM) incorporates spatial dependency into the OLS. The spatial error model (SEM) assumes spatial dependency in the error term.^(6)^

In the local models, the geographical context is considered, with estimates of specific parameters for each location.^(7)^ The geographically weighted regression (GWR) considers the spatial heterogeneity of data. A multiscale GWR considers variations in the studied relationships at different spatial scales.^(8)^

## II. Background

As of 22 June 2020, there were 1,106,470 confirmed COVID-19 cases in Brazil, which was at that point in time still the second leading country in the number of both total cases and total deaths (51,271).^(9)^

São Paulo is the largest city in Brazil, and also the largest city in Latin America, with a population of 12.3 million people.^(10)^ It plays an important role in the industrial and service sectors.^(11)^

There are important socioeconomic and demographic differences among the 96 districts of this mega-city (Map S1 and Table S1 in the online supplementary information). The central-western region is home to an elderly population with higher incomes per capita, while a younger population with a higher unemployment rate lives in the peripheral areas.^(12)^ The urban inequalities between the centre and the peripheral areas of São Paulo have been documented for over 20 years,^(13)^ and still constitute a problem to overcome. Budds and Teixeira indicated in 2005 that one-third of the inhabitants in the city of São Paulo lived in substandard settlements.^(14)^ Recently, Wilkinson has suggested that the population living in these types of settlements is more susceptible to COVID-19, because social isolation cannot be practised easily in the living environments that tend to accompany poor economic conditions.^(15)^

There are different types of informal settlements in São Paulo, with distinct features.^(16)^ A considerable number of informal settlements still present conditions of extreme deprivation, although there have been improvements in infrastructure during the 1990s and 2000s.^(17)^

It is important to note that a centre–periphery model is not capable of explaining the complexity of peripheral areas as there are spatial discontinuities caused by sub-centres, i.e. heterogeneous urban spaces constituting a sort of patchwork.^(18)^ This demonstrates the need for models to identify the relationship between socioeconomic spatial differences and the spread of diseases.

The socio-spatial differences in São Paulo are a concern for the city government, as undiagnosed cases occur mainly in the peripheral areas, and COVID-19 is expected to be underreported there.^(19)^ The Health Office of the city of São Paulo has been publishing fortnightly bulletins reporting the number of COVID-19 cases by district, as well as the number of deaths – both confirmed and suspected – to be due to COVID-19.^(20)^ At first glance, data show that the number of cases is higher in the central area, while the number of deaths is higher in the peripheral area, indicating the underreporting of COVID-19 cases in this area.

In general, in São Paulo, as in Brazil more generally, COVID-19 testing has been conducted primarily among hospitalized people,^(21)^ although wealthy people may also pay for precautionary testing.^(22)^ Therefore the numbers of confirmed and suspected deaths from COVID-19 seem a better representation of the situation in the city of São Paulo.

## III. Materials and Methods

Drawing on data made available by São Paulo State^(23)^ and on a digital map of the city of São Paulo,^(24)^ a list was composed of 18 demographic and socio-environmental explanatory variables. The following variables were considered: population (people); population density (people per square kilometre); population 60 years and older (people); population density of those 60 years and older (people per square kilometre); number of households per district; average number of people per household; number of informal urban settlements per district; illiteracy (%); graduates of higher education (%); average monthly income (R$ per capita); access to potable water (%); access to both a toilet and potable water (%); population living in a household with two or more people per bedroom (%); population living in an urban household with solid waste collection (%); population living in a household with access to electricity (%); population living in a household with inadequate sanitation (%); population living in substandard settlements (walls not made of brick or wood) (%); and the Municipal Human Development Index score.^(25)^

Test results for COVID-19 incidence fail to accurately represent the actual distribution in peripheral areas because access to diagnostic tests is more challenging there.^(26)^ This makes it difficult to compare rates in different parts of the city. Therefore, in an attempt to reduce this discrepancy, this study focused instead on the numbers of both confirmed and suspected deaths by COVID-19, which were analysed from 8 March to 18 June 2020.^(27)^

The Spearman correlation test at the 95 per cent significance level (α = 0.05) was used to assess the correlation between cumulative deaths (confirmed and suspected) and socio-environmental variables.

Seeking to understand the socio-spatial behaviour related to COVID-19 in the most impacted area in Brazil, five spatial regression^(28)^ models were analysed in order to assess the disease distribution in the affected territory. These included three global models – namely, the ordinary least squares (OLS), spatial error model (SEM) and spatial lag model (SLM) – and two local models – namely, geographically weighted regression (GWR) and multiscale geographically weighted regression (MGWR).^(29)^ The stepwise forward procedure^(30)^ and the Spearman correlation test were used to determine the most relevant explanatory variables for the regression models. The software programs GeoDa 1.14 and MGRW 2.2 were used to run the models, and the software program QGIS 3.10.4 was used for spatial analysis of the variables. The adjusted R² and the corrected Akaike Information Criterion (AICc) were used to analyse the performance of the models to explain death incidence in São Paulo.^(31)^

## IV. Results

Using the Spearman correlation test, significant positive correlations were found between deaths (confirmed and suspected as being due to COVID-19) and population (total and 60 years and older), population density (people per square kilometre), number of households, average people per household and informal urban settlements (Table 1).

**Table 1:** Spearman correlation coefficients: deaths (confirmed and suspected from COVID-19) vs. socio-environmental indicators, considering 96 districts with a total of 12,252,023 people

Social indicator (at district level)	Spearman correlation coefficient	*p*-value
Population (people)	**0.90**	0.00000*
60 years and older (people)	**0.80**	0.00000*
Population density (people/km^2^)	**0.41**	***0.00003***
Population density of 60 years and older (people/km^2^)	0.19	*0.06316*
Number of households	**0.85**	0.00000*
Average people per household	**0.46**	0.00000*
Number of informal urban settlements	**0.69**	0.00000*
Illiteracy (%)	0.09	*0.36422*
Graduates of higher education (%)	−0.15	*0.15163*
Average income (R$ per capita per month)	−0.16	*0.11825*
Access to potable water (%)	**–0.28**	***0.00590***
Access to toilet and potable water (%)	−0.03	*0.78638*
Household with two or more people per bedroom (%)	0.16	*0.12900*
Urban household with solid waste collection (%)	**–0.24**	***0.02000***
Household with access to electricity (%)	**–0.22**	***0.03418***
Household with inadequate sanitation (%)	0.17	*0.09198*
Substandard settlements (%)	0.01	*0.95909*
Municipal Human Development Index (MHDI)	−0.18	*0.08335*

NOTES:

R$: reais, Brazilian currency. US$ 1 = R$ 5.32, as of 10 September 2020.

In bold: significant correlation at α = 0.05.

* Less than 10^-6^.

Using both the stepwise forward procedure and the Spearman correlation test, the following explanatory variables were selected as the most relevant for the regression models: 60 years and older (people), population density (people per square kilometre), average people per household (people), and Municipal Human Development Index (MHDI), all at the district level.

In the OLS, SLM and SEM regression models, selected explanatory variables were positively associated with COVID-19 deaths (confirmed and suspected) (p-value < 0.01), except for the MHDI, indicating a weak relation of this index with the dependent variable. Because spatial dependence is incorporated into the SLM and SEM models, these regression models have shown slightly better performance than the OLS model (Table 2). Summary statistics of the three global models are presented in Tables S2 and S3 in the online supplement. The (M)GWR models have shown superior performance for data adjustment, as the AICc values were significantly lower than the values obtained for the global models (Table 2).

**Table 2:** Regression parameters for model selection to explain death incidence by COVID-19 in São Paulo

	OLS	SLM	SEM	GWR	MGWR
**Adjusted R²**	0.857	0.865	0.869	0.900	0.899
**AICc**	882.27	882.68	879.41	78.76	79.81

Overall, the adjusted R² values indicate that selected explanatory variables explain between 85.7 per cent and 90.0 per cent of the COVID-19 death incidence (confirmed and suspected).

**MAP 1:**
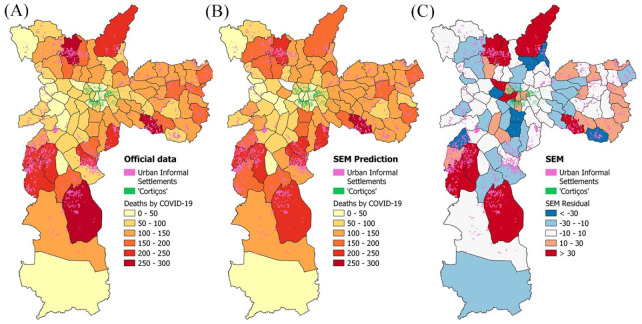
Geographic distribution of death incidence by COVID-19 reported cases (1A), COVID-19 SEM-predicted cases (1B), and residuals (real – prediction) (1C) in São Paulo

Considering the global models, and analysing both the R² and the AICc, the SEM model has presented best adherence to analysed data (Table 2). A map of reported COVID-19 cases, SEM-predicted COVID-19 cases, and residuals (real – prediction) is presented in Maps 1A–1C. The highest residuals were found: i) to be positive values in the districts with more reported deaths and in districts in the historic centre; and ii) to be negative values in districts with low death incidence compared to their neighbours. Moreover, using the overlay procedure to show the locations of informal urban settlements, we have observed that the districts with the most deaths are those with higher numbers of informal urban settlements.

**MAP 2:**
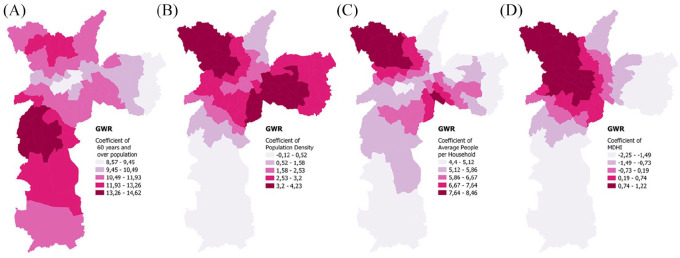
The effects of people aged 60 years and older (2A), population density (2B), average people per household (2C) and MDHI (2D) in describing death incidence by COVID-19 in São Paulo using the GWR model

The models based on geographically weighted regression (GWR) provide a spatial evaluation of distinct variables, as the process spatial heterogeneity is considered,^(32)^ unlike the global models. Although the multiscale variation (M) of the GWR model is expected to display better outputs,^(33)^ our results show comparable performances of the GWR and MGWR models. Because the GWR is more suitable for a detailed assessment at the district scale, the GWR model was used to analyse the selected variables influencing the spread of COVID-19 in São Paulo. Spatial variations of the selected variables can be detected by analysis of their coefficients using the GWR model (Maps 2A–2D).

In the northwestern region of São Paulo, the population density, average people per household and MDHI variables represent well the number of deaths, while in the southwestern region of the city, the number of people aged 60 years and older proved to influence the number of deaths in densely populated districts with numerous informal urban settlements. In the central area of the city the MHDI is inversely related to the number of deaths.

## V. Discussion

Our results have demonstrated that the GWR model best explained the spatial distribution of COVID-19 in the city of São Paulo, highlighting the spatial aspects of the data. Mollalo et al. selected the MGWR as the best model for data from the United States (R² = 0.68).^(34)^ Because the spatial heterogeneity is considered in the local models in order to evaluate the influence of each variable in the final output, a more detailed analysis is possible. Hence, we suggest a preliminary analysis of the models when feasible, or the conjunction of global and local models to better understand the spread of the COVID-19 and also other infectious diseases in large cities.

Analysing the number of deaths (confirmed and suspected) is imperative to understand the spread of COVID-19 in the city of São Paulo. In Brazil, even in wealthier cities, the lack of testing for COVID-19 has been an issue,^(35)^ and diagnosis may be especially difficult among disadvantaged people.^(36)^ For example, the district with the highest number of confirmed cases on 30 April 2020 had 200 cases and 11 deaths, while the district with the highest number of deaths had 103 deaths and 130 confirmed cases. This discrepancy probably occurs due to access to paid tests for the population with high incomes, indicating the underreporting in peripheral areas. Furthermore, in Brazil, the vulnerability of the health system is also a reason for concern. Hospital beds, for example, are inefficiently distributed,^(37)^ and support measures for vulnerable populations are inadequate. It is also important to emphasize the effectiveness of social isolation measures in the metropolitan area of São Paulo as a means of controlling the load of COVID-19 in the healthcare system, especially the public system. One recent study reported that the social isolation rate^(38)^ was found to be negatively correlated with cumulative confirmed COVID-19 cases.^(39)^

The studied models fit very well (R² greater than 0.85) real data on confirmed and suspected deaths due to COVID-19 in the city of São Paulo. However, death incidence per 1,000 people is high in some districts in the historic centre (Table S1 in the online supplement), although the number of deaths is lower than in other districts. The GWR model explicitly shows this discrepancy, which is also evidenced by the MDHI’s influence (Map 2). Households known as *cortiços*^(40)^ in the historic centre of the city of São Paulo present significant differences compared to nearby districts because of their precarious conditions and high social vulnerability.^(41)^

Results obtained using the Spearman correlation test have made evident the correlation between COVID-19 death incidence and social aspects such as population density, average people per household, and informal urban settlements. Spatial analysis has shown the spread of COVID-19 in areas with highly vulnerable populations (Map 1). Recently, Corburn et al. and Wilkinson have reported that social distancing and self-quarantine may be impractical for vulnerable populations, because of the following factors: i) high numbers of informal workers, leading to financial vulnerability; ii) space constraints; iii) inadequate access to water and sanitation; and iv) a lack of secure and adequate housing.^(42)^ Furthermore, it is worth mentioning a cultural feature that may also influence the adherence to social distancing: the Brazilian *jeitinho*, described as a social mechanism of breaking rules to deal with unexpected situations.^(43)^ Therefore, governmental^(44)^ and social^(45)^ initiatives^(46)^ have sought to communicate better with the population in São Paulo as a means of improving the awareness of the importance of the social isolation measures and thus increase the adherence to social distancing.

Our results are consistent with vulnerability aspects reported by Wilkinson, notably population density and household conditions.^(47)^ People aged 60 years and older have proven to be a determining variable influencing the spread of COVID-19, corroborating data presented by Lloyd-Sherlock.^(48)^ Even though informal settlements in São Paulo present a variety of conditions, the average number of people per household is similar to the number of people in formal households, while the population density in areas of informal settlements is two–five times higher than in areas of formal households.^(49)^

## VI. Conclusions

In this study, we have used spatial modelling to assess the spread of COVID-19 in a metropolitan area in the global South. All analysed spatial regression models, using four selected explanatory variables, have proven to represent at least 85 per cent of reported deaths at the district level. Overall, the local model GWR has shown the best adjustment to the analysed data (R² = 0.900, AICc = 78.76). More importantly, our study has revealed a significant correlation between COVID-19 death incidence and social aspects such as population density, people aged 60 years and older, and informal urban settlements. Our findings have shown that the high population density found in informal settlements in the city of São Paulo is a determining factor influencing the spread of COVID-19, possibly because people living in socioeconomic vulnerability may not be able to adhere to social distancing measures. Our study corroborates reports from the recent literature, pointing to the need for special attention in peripheral areas and informal settlements.

## Supplemental Material

urban-supplement_1 – Supplemental material for GIS-based spatial modelling of COVID-19 death incidence in São Paulo, Brazil Click here for additional data file. Supplemental material, urban-supplement_1 for GIS-based spatial modelling of COVID-19 death incidence in São Paulo, Brazil by Rodrigo Custodio Urban and Liane Yuri Kondo Nakada in Environment & Urbanization

## Footnotes

1. WHO (2020), *WHO Director-General’s Opening Remarks at the Media Briefing on COVID19 - 11 March 2020*, accessed 25 April 2020 at https://www.who.int/dg/speeches/detail/who-director-general-s-opening-remarks-at-the-media-briefing-on-covid-19—11-march-2020.

2. Worldometer (2020), *Coronavirus*, accessed 13 May 2020 at https://www.worldometers.info/coronavirus.

3. SEADE (2020a), *Coronavirus*, accessed 10 May 2020 at https://www.seade.gov.br/coronavirus.

4. Mollalo, A, B Vahedi and K M Rivera (2020), “GIS-based spatial modeling of COVID-19 incidence rate in the continental United States”, *Science of the Total Environment* Vol 728, accessed 10 September 2020 at https://doi.org/10.1016/j.scitotenv.2020.138884.

5. See reference 4; also Brundson, C, S Fotheringham and M Charlton (2002), “Geographically weighted regression”, *Journal of the Royal Statistical Society: Series D (The Statistician)* Vol 47, No 3, accessed 10 September 2020 at https://doi.org/10.1111/1467-9884.00145.

6. See reference 4.

7. Oshan, T M, Z Li, W Kang, L J Wolf and A S Fotheringham (2019), “Mgwr: a Python implementation of multiscale geographically weighted regression for investigating process spatial heterogeneity and scale”, *ISPRS International Journal of Geo-Information* Vol 8, No 6, accessed 10 September 2020 at https://doi.org/10.3390/ijgi8060269.

8. See reference 7.

9. See reference 2.

10. See reference 3.

11. SEADE (2019), *São Paulo 465 Anos*, accessed 12 May 2020 at https://www.seade.gov.br/wp-content/uploads/2019/02/Municipio_Sao_Paulo_.pdf.

12. See reference 11.

13. Stephens, C, M Akerman, S Avle, P B Maia, P Campanario, B Doe and D Tetteh (1997), “Urban equity and urban health: using existing data to understand inequalities in health and environment in Accra, Ghana and São Paulo, Brazil”, *Environment and Urbanization* Vol 9, No 1, pages 89–113; also Marques, E and C Saraiva (2017), “Urban integration or reconfigured inequalities? Analyzing housing precarity in São Paulo, Brazil”, *Habitat International* Vol 69, pages 18–26, accessed 10 September 2020 at https://doi.org/10.1016/j.habitatint.2017.08.004.

14. Budds, J and P Teixeira (2005), “Ensuring the right to the city: pro-poor housing, urban development and tenure legalization in São Paulo, Brazil”, *Environment and Urbanization* Vol 17, No 1, pages 89–113.

15. Wilkinson, A (2020), “Local response in health emergencies: key considerations for addressing the COVID-19 pandemic in informal urban settlements”, *Environment and Urbanization* Vol 32, No 2, pages 503–522, accessed 10 September 2020 at https://doi.org/10.1177/0956247820922843.

16. See reference 13, Marques and Saraiva (2017).

17. See reference 13, Marques and Saraiva (2017); also Torres, H da G, E C L Marques and R M Bichir (2006), “Políticas públicas, pobreza urbana e segregação residencial”, in J M P da Cunha (editor), *Novas Metrópoles Paulistas: População, Vulnerabilidade e Segregação*, Núcleo de Estudos de População/UNICAMP, Campinas; and Caldeira, T P R (2016), “Peripheral urbanization: autoconstruction, transversal logics, and politics in cities of the global South”, *Environmental and Planning D: Society and Space* Vol 35, No 1, pages 3–20, accessed 10 September 2020 at https://doi.org/10.1177/0263775816658479.

18. See reference 17, Caldeira (2016); also Torres, H da G, E Marques, M P Ferreira and S Bitar (2003), “Pobreza e espaço: padrões de segregação em São Paulo”, *Estudos Avançados* Vol 17, No 47, pages 97–128, accessed 10 September 2020 at https://doi.org/10.1590/S0103-40142003000100006.

19. SMS-SP (2020a), *COVID-19 Boletim Quinzenal*, 54 pages, accessed 5 May 2020 at https://www.prefeitura.sp.gov.br/cidade/secretarias/upload/saude/PMSP_SMS_COVID19_Boletim%20Quinzenal_20200430.pdf.

20. SMS-SP (2020b), *Boletins COVID-19*, accessed 14 May 2020 at https://www.prefeitura.sp.gov.br/cidade/secretarias/saude/vigilancia_em_saude/doencas_e_agravos/coronavirus/index.php?p=295572.

21. Barifouse, R (2020), “Coronavírus: por que o Brasil ainda não conseguiu fazer testes em massa?”, *BBC News*, 3 April, accessed 14 June 2020 at https://www.bbc.com/portuguese/brasil-52145795; also Figueiredo, P (2020), “Bairros com maior número de mortes por coronavirus em SP concentram favelas e conjuntos habitacionais”, *G1*, accessed 8 May 2020 at https://g1.globo.com/sp/sao-paulo/noticia/2020/05/04/bairros-com-maior-numero-de-mortes-por-coronavirus-em-sp-concentram-favelas-e-conjuntos-habitacionais.ghtml.

22. See reference 19; also see reference 21, Figueiredo (2020).

23. SEADE (2020b), *IMP*, accessed 18 June 2020 at https://www.imp.seade.gov.br.

24. São Paulo (2020a), *Mapa Digital da Cidade de São Paulo*, accessed 5 May 2020 at http://geosampa.prefeitura.sp.gov.br/PaginasPublicas/_SBC.aspx.

25. The Municipal Human Development Index (MHDI) is an adaptation of the Human Development Index (HDI) to Brazilian cities, considering regional challenges for human development. Like the HDI, the MHDI varies from 0 to 1 and is based on health, education and income; however, it uses different indicators. [PNUD/IPEA/FJP (2013), *Índice de Desenvolvimento Humano Municipal Brasileiro*, Brasília, 96 pages, acessed 28 August 2020 at http://www.atlasbrasil.org.br/2013/data/rawData/publicacao_atlas_municipal_pt.pdf.])

26. See reference 19.

27. See reference 20.

28. More detailed information about the models is available in Ward, M D and K S Gleditsch (2018), *Spatial Regression Models*, Sage Publishing, 128 pages; Oshan et al. (see reference 7); and Mollalo et al. (see reference 4).

29. See reference 4.

30. See reference 4.

31. See reference 4.

32. See reference 7.

33. See reference 7.

34. See reference 4.

35. Barrucho, L (2020), “Brasil é um dos países que menos realiza testes para covid-19, abaixo de Cuba e Chile”, *BBC News*, 24 April, accessed 29 April 2020 at https://www.bbc.com/portuguese/internacional-52383539.

36. See reference 21, Figueiredo (2020).

37. Requia, W J, E K Kondo, M D Adam, D R Gold and C J Struchinere (2020), “Risk of the Brazilian health care system over 5572 municipalities to exceed health care capacity due to the 2019 novel coronavirus (COVID-19)”, *Science of the Total Environment* Vol 730, accessed 10 September 2020 at https://doi.org/10.1016/j.scitotenv.2020.139144.

38. The social isolation rate represents a daily estimate of the proportion of the population not moving further than 200 metres from the location of their mobile phone (indicating their home location) during the previous night. These are official data made available by the São Paulo state government. [IPT (2020), *Perguntas sobre Isolamento Social: IPT Responde Perguntas Frequentes (FAQs) sobre Índices de Isolamento Social Divulgados pelo Governo de SP*, accessed 2 September 2020 at https://www.ipt.br/noticia/1623-_perguntas_sobre_isolamento_social.htm; also São Paulo (2020b), *São Paulo State – Social Isolation Intelligent Monitoring System*, accessed 28 August 2020 at https://www.saopaulo.sp.gov.br/coronavirus/isolamento.]

39. Nakada, L Y K and R C Urban (2020, in press), “COVID-19 pandemic: environmental and social factors influencing the spread of SARS-CoV-2 in São Paulo, Brazil”, *Environmental Science and Pollution Research* at https://doi.org/10.1007/s11356-020-10930-w.

40. *Cortiço* is precarious rental housing.

41. See reference 13, Marques and Saraiva (2017).

42. See reference 15; also Corburn, J, D Vlahov, B Mberu, L Riley, W T Caiaffa, S F Rashid, A Ko, S Patel, S Jukur, E Martínez-Herrera, S Jayasinghe, S Agarwal, B Nguendo-Yongsi, J Weru, S Ouma, K Edmundo, T Oni and H Ayad (2020), “Slum health: arresting COVID-19 and improving well-being in urban informal settlements”, *Journal of Urban Health*, accessed 10 September 2020 at https://doi.org/10.1007/s11524-020-00438-6.

43. Duarte, F (2006), “Exploring the interpersonal transaction of the Brazilian jeitinho in bureaucratic contexts”, *Organization* Vol 13, No 4, pages 509–527, accessed 10 September 2020 at https://doi.org/10.1177/1350508406065103; also Ferreira, M C, R Fischer, J B Porto, R Pilati and T L Milfont (2012), “Unraveling the mystery of Brazilian jeitinho: a cultural exploration of social norms”, *Personality and Social Psychology Bulletin* Vol 38, No 3, pages 331–344, accessed 10 September 2020 at https://doi.org/10.1177/0146167211427148; and Miura, M A, R Pilati, T L Milfont, M C Ferrreira and R Fischer (2019), “Between simpatia and malandragem: Brazilian jeitinho as an individual difference variable”, *PLoS ONE* Vol 14, No 4, accessed 10 September 2020 at https://doi.org/10.1371/journal.pone.0214929.

44. SMS-SP (2020c), *Equipes de Saúde Atuam na Prevenção da Covid-19 nas Áreas Mais Vulneráveis da Capital*, acessed 16 June 2020 at https://www.prefeitura.sp.gov.br/cidade/secretarias/saude/noticias/index.php?p=296667.

45. Vilhena, E and V Guilhermina (2020), “ ‘Não estamos no mesmo barco’: periferias de SP adaptam ações contra Covid”, *UOL*, 3 June, accessed 16 June 2020 at https://www.uol.com.br/ecoa/ultimas-noticias/2020/06/03/periferias-de-sp-criam-acoes-atentas-a-condicoes-locais-para-prevenir-covid.htm?cmpid=copiaecola.

46. These initiatives include healthcare personnel visits, as well as informative podcasts, factsheets and loudspeaker car messages created by individuals from peripheral areas or living in informal settlements.

47. See reference 15.

48. Lloyd-Sherlock, P, S B Ebrahim, L Geffen and M McKee (2020), “Bearing the brunt of covid-19: older people in low and middle income countries”, *BMJ* Vol 368, accessed 10 September 2020 at https://doi.org/10.1136/bmj.m1052.

49. See reference 13, Marques and Saraiva (2017).
