# Supplementary material for: GIS-based spatial modelling of COVID-19 death incidence in São Paulo, Brazil
Source: Environ Urban. 2021 Apr;33(1):229–38. doi: 10.1177/0956247820963962 (PMC7557234; doi:10.1177/0956247820963962)
Supplement: urban-supplement_1 – Supplemental material for GIS-based spatial modelling of COVID-19 death incidence in São Paulo, Brazil [file urban-supplement_1.pdf]

GIS-based spatial modelling of COVID-19 death incidence in São Paulo, Brazil

Online supplementary information

MAP S1
Districts of the city of São Paulo

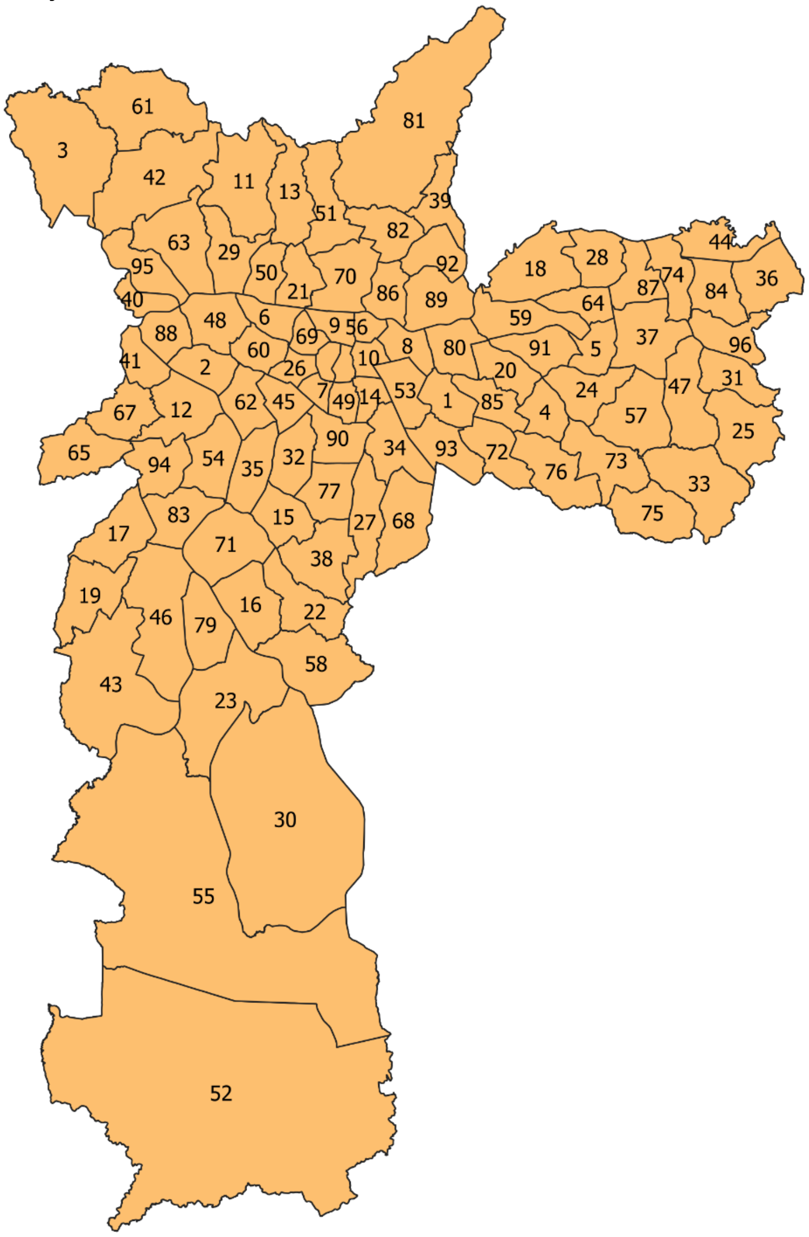

TABLE S1
Summary characteristics of the districts of the city of São Paulo and COVID-19 deaths (confirmed and suspected), as of 18 June 2020

Code	District	Population	COVID-19 deaths	Deaths per 1,000 people
1	Água Rasa	82,564	111	1.20
2	Alto de Pinheiros	40,962	44	0.94
3	Anhanguera	84,719	36	0.50
4	Aricanduva	85,868	91	0.93
5	Artur Alvim	100,462	139	1.21
6	Barra Funda	16,115	19	1.21
7	Bela Vista	73,235	37	0.49
8	Belém	49,213	69	1.41
9	Bom Retiro	38,877	37	1.00
10	Brás	33,045	45	1.41
11	Brasilândia	281,977	277	0.96
12	Butanta	53,836	41	0.69
13	Cachoeirinha	146,387	178	1.14
14	Cambuci	40,667	44	1.09
15	Campo Belo	63,744	85	1.19
16	Campo Grande	106,722	88	0.80
17	Campo Limpo	228,893	144	0.63
18	Cangaiba	138,107	153	1.03
19	Capao Redondo	296,378	237	0.81
20	Carrao	84,925	94	1.04
21	Casa Verde	86,004	108	1.16
22	Cidade Ademar	285,677	225	0.77
23	Cidade Dutra	203,131	159	0.74
24	Cidade Lider	135,247	126	0.91
25	Cidade Tiradentes	235,630	193	0.84
26	Consolacao	57,405	52	0.83
27	Cursino	113,728	117	0.99
28	Ermelino Matarazzo	118,715	106	0.86
29	Freguesia do O	140,083	181	1.17
30	Grajau	390,096	267	0.68
31	Guaianases	109,730	124	0.69
32	Iguatemi	149,739	137	0.99
33	Ipiranga	112,222	115	0.99
34	Itaim Bibi	97,229	90	0.89
35	Itaim Paulista	234,912	173	0.71
36	Itaquera	211,555	187	0.84
37	Jabaquara	229,346	199	0.82
38	Jaçanã	96,054	84	0.82
39	Jaguara	23,950	19	0.70
40	Jaguaré	55,192	46	0.85
41	Jaraguá	212,819	142	0.71
42	Jardim Ângela	338,265	240	0.75
43	Jardim Helena	135,605	149	1.01
44	Jardim Paulista	90,719	64	0.66
45	Jardim São Luís	293,660	235	0.81

46	José Bonifácio	136,560	117	0.87
47	Lajeado	174,539	161	1.42
48	Lapa	67,170	62	0.87
49	Liberdade	72,797	73	0.97
50	Limão	79,657	115	1.32
51	Mandaqui	109,228	116	0.99
52	Marsilac	8,426	9	1.00
53	Moema	89,382	66	0.73
54	Moóca	80,330	91	1.10
55	Morumbi	52,921	38	0.74
56	Parelheiros	153,598	113	0.79
57	Pari	19,069	27	1.43
58	Parque do Carmo	71,749	69	0.93
59	Pedreira	160,976	92	0.59
60	Penha	129,100	128	0.92
61	Perdizes	114,788	87	0.72
62	Perus	89,310	76	0.87
63	Pinheiros	65,909	53	0.74
64	Pirituba	171,232	149	0.81
65	Ponte Rasa	89,774	102	1.00
66	Raposo Tavares	107,426	88	0.81
67	República	61,832	64	1.03
68	Rio Pequeno	123,711	98	0.76
69	Sacomã	263,621	226	0.84
70	Santa Cecília	88,518	91	1.00
71	Santana	113,253	128	0.99
72	Santo Amaro	74,447	81	1.04
73	São Domingos	86,403	85	0.92
74	São Lucas	142,948	146	0.94
75	São Mateus	155,387	168	0.99
76	São Miguel	89,173	112	1.12
77	São Rafael	159,683	95	0.61
78	Sapopemba	289,759	300	0.97
79	Saúde	134,147	112	0.79
80	Sé	26,693	25	0.97
81	Socorro	36,033	33	0.80
82	Tatuapé	96,045	100	1.00
83	Tremembé	223,553	218	1.02
84	Tucuruvi	96,358	94	0.88
85	Vila Andrade	163,508	86	0.62
86	Vila Curuçá	153,500	141	0.87
87	Vila Formosa	94,100	106	1.03
88	Vila Guilherme	57,079	64	1.08
89	Vila Jacuí	145,836	138	0.89
90	Vila Leopoldina	45,092	27	0.63
91	Vila Maria	114,025	111	0.90
92	Vila Mariana	132,226	112	0.79
93	Vila Matilde	105,575	112	0.98
94	Vila Medeiros	123,456	153	1.08
95	Vila Prudente	104,686	121	1.07

96	Vila Sônia	121,663	67	0.57

SOURCE: SEADE (2020), IMP, accessed 18 June 2020 at https://www.imp.seade.gov.br.

TABLE S2
Summary statistics of the OLS model for selected variables in modelling COVID-19 deaths in the city of São Paulo

**Variable**	**Coefficient**	**Std. Error**	**T-statistic**	**P-value**
Intercept	-212.49700	53.32850	-3.98467	0.00014
Population aged 60+	0.0051528	0.000298	17.29070	0.00000
Average people per household	85.1767	9.24437	9.21391	0.00000
Population density	0.0018544	0.0005156	3.59679	0.00052
MHDI	-50.4409	48.8182	-1.03324	0.30423

Table S3
Summary statistics of SLM and SEM in modelling COVID-19 deaths in the city of São Paulo

**Variable**	**Coefficient**		**Std. error**		***Z*-score**		**P-value**	
	**SLM**	**SEM**	**SLM**	**SEM**	**SLM**	**SEM**	**SLM**	**SEM**
Intercept	-201.896	-209.452	52.0743	53.3810	-3.8771	-3.9237	0.0001	0.0001
Pop_60+	0.0050	0.0053	0.0003	0.0003	15.7863	17.0281	0.0000	0.0000
Average people/household	78.0022	80.3288	10.2299	10.5741	7.6249	7.5968	0.0000	0.0000
Population density	0.0018	0.0014	0.0005	0.0006	3.6384	2.5619	0.0003	0.0104
MHDI	-47.9395	-34.0116	47.0889	46.3686	-1.0181	-0.7335	0.3087	0.4633
Rho	0.0994	–	0.0763	–	1.3024	–	0.1928	–
Lambda	–	0.2880	–	0.1348	–	2.1362	–	0.0327
